# Genome sequences of two closely related strains of *Escherichia coli* K-12 GM4792

**DOI:** 10.1186/s40793-015-0114-x

**Published:** 2015-12-10

**Authors:** Yan-Cong Zhang, Yan Zhang, Bi-Ru Zhu, Bo-Wen Zhang, Chuan Ni, Da-Yong Zhang, Ying Huang, Erli Pang, Kui Lin

**Affiliations:** State Key Laboratory of Earth Surface Processes and Resource Ecology and MOE Key Laboratory for Biodiversity Science and Ecological Engineering, College of Life Sciences, Beijing Normal University, 19 Xinjiekouwai Street, Beijing, 100875 China; State Key Laboratory for Infectious Disease Prevention and Control, and National Institute for Communicable Disease Control and Prevention, Chinese Center for Disease Control and Prevention, Beijing, 102206 China; Present address: National Laboratory of Biomacromolecules, Institute of Biophysics, Chinese Academy of Sciences, Beijing, 100101 China; Present address: The second high school attached to Beijing Normal University, Beijing, 100192 China

**Keywords:** *Escherichia coli* K12, GM4792, Lactose, Gram-negative, Genome comparison, Experimental evolution, Variant analysis

## Abstract

*Escherichia coli* lab strains K-12 GM4792 Lac^+^ and GM4792 Lac^-^ carry opposite lactose markers, which are useful for distinguishing evolved lines as they produce different colored colonies. The two closely related strains are chosen as ancestors for our ongoing studies of experimental evolution. Here, we describe the genome sequences, annotation, and features of GM4792 Lac^+^ and GM4792 Lac^-^. GM4792 Lac^+^ has a 4,622,342-bp long chromosome with 4,061 protein-coding genes and 83 RNA genes. Similarly, the genome of GM4792 Lac^-^ consists of a 4,621,656-bp chromosome containing 4,043 protein-coding genes and 74 RNA genes. Genome comparison analysis reveals that the differences between GM4792 Lac^+^ and GM4792 Lac^-^ are minimal and limited to only the targeted *lac* region. Moreover, a previous study on competitive experimentation indicates the two strains are identical or nearly identical in survivability except for lactose utilization in a nitrogen-limited environment. Therefore, at both a genetic and a phenotypic level, GM4792 Lac^+^ and GM4792 Lac^-^, with opposite neutral markers, are ideal systems for future experimental evolution studies.

**Electronic supplementary material:**

The online version of this article (doi:10.1186/s40793-015-0114-x) contains supplementary material, which is available to authorized users.

## Introduction

The microbial experimental evolution systems, with the ability to generate a ‘fossil’ record for later study and the design of replicate populations to test the predictability of evolution, offer a chance to ‘replay’ the evolutionary process, ‘watch’ evolution in action [1] and measure the fitness of evolved lines under the relevant environmental conditions [2]. However, the lack of obvious differences in phenotypic characteristics makes microbes difficult to observe. Fortunately, some neutral genetic markers help distinguish evolved lines by differences in colony color [2]. Typically, when a derived strain with an opposite marker relative to its progenitor is required, one can be selected using specific culture media [3]. Subsequently, the degree of neutrality for this marker is evaluated by comparing the fitness of the two strains containing opposite markers under the culture conditions used in the study [4]. The lactose marker is one such marker. For the *lac* operon, a previous study has been performed utilizing its mutations between strains with opposite lactose markers via target sequencing [5].

Since the publication of the K-12 genome in 1977 [6], *Escherichia coli* has been thoroughly studied with regard to its genetics [7–9], biochemistry [10–12], metabolic reconstruction [10], pathway inference [13], genomics [14–16] and metabolic [17]. *E. coli* strain K-12 GM4792, a laboratory strain, contains the chromosomal *lacI33::lacZ* allele and is unable to utilize lactose [18]. GM4792 was a derivative of the parent strain P90C [*ara-600* del(*gpt-lac*)5 LAM^-^*relA1 spoT1thiE1*] [19–21] by homogenizing a Pro^+^ Lac^+^/F' *lacI33::lac*Z and then curing the episome with acridine orange [20] (M. G. Marinus, personal communication). A previous study [22] resulted in two closely related strains, GM4792 Lac^-^ and GM4792 Lac^+^ that carry opposite lactose markers and plasmids are knocked out for further studies on experimental evolution. Here, Lac^+^ refers to the ability of the strain to utilize lactose and Lac^-^ refers to the inability to utilize lactose. These strains were chosen as ancestors for our ongoing studies of the experimental evolution of *E. coli* in a nitrogen-limited environment. In this study, we summarize the classification and features of *E. coli* GM4792 Lac^+^ and GM4792 Lac^-^, together with a description of the genome sequencing and annotation. This work provides a foundation for future variant analysis of evolved lines at the genomic scale. To compare GM4792 Lac^+^ and GM4792 Lac^-^, we used the *breseq* pipeline v0.20 [23] to detect initial variants and subsequently applied a series of filters to eliminate false positives. Using this method, two significant variants were detected, including a synonymous single nucleotide polymorphism, and a 1-bp deletion responsible for lactose metabolism. A previous study on competitive experimentation [22] has shown that these two strains are identical or nearly identical in survivability, except for lactose utilization in a nitrogen-limited environment. Thus, both genetically and phenotypically, GM4792 Lac^+^ and GM4792 Lac^-^ carry neutral markers and are appropriate for future experimental evolution studies.

## Organism information

### Classification and features

**Fig. 1:**
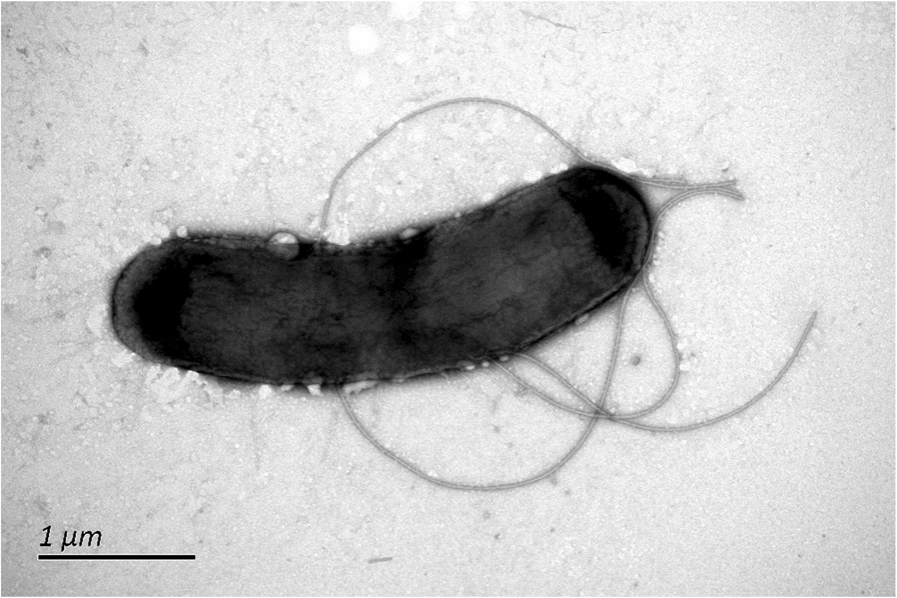
Scanning-electron micrograph of strain *E. coli* GM4792 Lac^+^

GM4792 is a strain of *E. coli* K-12. It is asexual (F^-^), carries *lacI33::lacZ* allele and cannot metabolize lactose [18]. This laboratory strain was a generous gift from M. G. Marinus (University of Massachusetts Medical School). We obtained it on October 7, 2007. Firstly, GM4792 was transferred to Luria-Bertani (LB) liquid medium for 24 h with shaking at 150 rpm. Subsequently, strains were streaked on LB solid medium. Twenty-four hours later, a single colony was transferred to LB liquid medium, with shaking for 24 h. The inoculated medium was mixed 1:1 with glycerol saline and stored in a –40 °C freezer. Thus, a monoclonal GM4792 Lac^-^ strain was obtained. The monoclonal GM4792 Lac^-^ colonies were grown in LB liquid medium, collected by centrifugation, and washed with the culture solution. Then, approximately 10^9^ cells were plated on Davis minimal media [4] containing only lactose as the carbon source. Following a 4-day incubation period, the colonies began to utilize the lactose in the medium. One colony was selected and amplified in LB liquid medium, then stored at –40 °C. Thus, GM4792 Lac^+^ strain was obtained with the ability to metabolize lactose. The genome of each strain is a single circular chromosome with knockout plasmids; so, genetic variants between them could arise only from *de novo* mutations. Like most strains of *E. coli* [7], the cells of GM4792 are rod-shaped (Fig. 1, Additional file 1: Figure S1), Gram-negative, motile with peritrichous flagella, non-pigmented, chemo-organotrophic and facultative anaerobes. As GM4792 does not ferment sucrose or salicin, the strain belongs to *E. coli**“var. communis”* [24]. As previously described, GM4792 can grow at temperatures between 10 °C and 45 °C, with an optimum growth temperature of 37 °C, and pH 5.5-8.0 [25, 26]. Strain characteristics of *E. coli* K-12 GM4792 are shown in Table 1.

**Table 1:** Classification and general features of *Escherichia coli* strain K-12 GM4792 according to the MIGS recommendations [58]

MIGS ID	Property	Term	Evidence code^a^
	Classification	Domain *Bacteria*	TAS [59]
		Phylum *Proteobacteria*	TAS [60]
		Class *Gammaproteobacteria*	TAS [61]
		Order *Enterobacteriales*	TAS [61, 62]
		Family *Enterobacteriaceae*	TAS [63]
		Genus *Escherichia*	TAS [64, 65]
		Species *Escherichia coli*	TAS [64, 65]
		Strain: GM4792	TAS [22]
	Gram stain	Negative	IDA, TAS [25]
	Cell shape	Rod	TAS [25]
	Motility	Motile	TAS [25]
	Sporulation	None	IDA, TAS [25]
	Temperature range	10 °C ~ 45 °C	NAS
	Optimum temperature	37 °C	IDA, TAS [66]
	pH range; Optimum	5.5–8.0; 7	IDA, TAS [25, 26]
	Carbon source	peptides	IDA, TAS [66]
MIGS-6	Habitat	Not reported	
MIGS-6.3	Salinity	Not reported	
MIGS-22	Oxygen requirement	Facultative anaerobe	TAS [25, 26]
MIGS-15	Biotic relationship	Human specimen	NAS
MIGS-14	Pathogenicity	Non-pathogenic	NAS
MIGS-4	Geographic location	Not reported	
MIGS-5	Sample collection	October 7, 2007	
MIGS-4.1	Latitude	Not reported	
MIGS-4.2	Longitude	Not reported	
MIGS-4.4	Altitude	Not reported	

^a^Evidence codes - IDA: Inferred from Direct Assay; TAS: Traceable Author Statement (i.e., a direct report exists in the literature); NAS: Non-traceable Author Statement (i.e., not directly observed for the living, isolated sample, but based on a generally accepted property for the species, or anecdotal evidence). These evidence codes are from the Gene Ontology project [46]. Some missing key taxonomic references are shown in Additional file 3

As a model organism, the molecular structure and chemical composition of the cell wall of *E. coli* have been thoroughly studied. This is described in detail by Scheutz and Strockbine [26]. Similar to other strains of *E. coli*, GM4792 has a single peptidoglycan layer within the periplasm, consisting of D-glutamic acid, D-alanine, mesodiaminopimelic acid, N-acetyglucosamine and N-acetylmuramic acid linked to the tetrapeptide L-alanine. The cells stain Gram-negative and contain an outer membrane, with a lipopolysaccharide layer containing lipid A, the core region of the phosphorylated nonrepeating oligosaccharides and the O-antigen polymer [7, 25, 26].

## Genome sequencing information

### Genome project history

The two closely related *E. coli* lab strains K-12 GM4792 Lac^+^ and GM4792 Lac^-^ were selected for genome sequencing for subsequent use in experimental evolution studies. The genomes were sequenced in the year 2012. The genome project is deposited at the Genome OnLine Database [27] and the NCBI BioProject database. The finished genome sequences are deposited at GenBank with the accession numbers CP011342 and CP011343. A summary of the project information is shown in Table 2.

**Table 2:** Project information

MIGS ID	Property	Term
MIGS 31	Finishing quality	High-quality draft
MIGS-28	Libraries used	Two paired-end libraries of 180 bp, 380 bp and two mate-pair libraries of 2,000 bp, 6,000 bp, respectively
MIGS 29	Sequencing platforms	Illumina HiSeq 2000
MIGS 31.2	Fold coverage	~330× for GM4792 Lac^+^ and ~370× for GM4792 Lac^-^ (180 bp); ~100x (other libraries)
MIGS 30	Assemblers	ALLPATHS-LG Release 42411 [31]
MIGS 32	Gene calling method	RATT, Prodigal v2.5 [35]
	Locus Tag	U068 for Lac^+^ and U069 for Lac^-^
	Genbank ID	CP011342 for Lac^+^ and CP011343 for Lac^-^
	GenBank Date of Release	Jun 6, 2015
	GOLD ID	Gi0059689 for GM4792 Lac^+^ and Gi0059688 for GM4792 Lac^-^
	BIOPROJECT	PRJNA224130 for GM4792 Lac^+^ and PRJNA224131 for GM4792 Lac^-^
	SRA IDs	GM4792 Lac^+^ : SRR2596368, SRR2537294,
		SRR2619692, SRR2619693
		GM4792 Lac^-^ : SRR2529478, SRR1039666,
		SRR2529494, SRR2533204
MIGS 13	Source Material Identifier	GM4792
	Project relevance	Experimental evolution, Tree of Life

### Growth conditions and genomic DNA preparation

After receiving the laboratory strain GM4792 from M. G. Marinus, a single clone was randomly selected as a Lac^-^ strain. A single Lac^+^ clone was obtained after the Lac^-^ strain had been incubated for 4 days under selection conditions for lactose metabolism. Strains stored at –40 °C were thawed at room temperature. Each strain was streaked on LB solid medium with an inoculation needle and incubated for 24 h at 37 °C. Distinctive monoclonal colonies grew, and a single colony was selected and inoculated into 5 ml LB liquid medium and grown at 37 °C with shaking for 24 h. Total genomic DNA was extracted using the TIANamp Bacteria DNA Kit (Code:DP302, TIANGEN BIOTECH, Beijing, China), according to the manufacturer’s instructions. Additional RNaseA (Code:RT405-12, TIANGEN BIOTECH CO, Beijing, China) was added, following the manufacturer’s instruction. The quality and quantity of the genomic DNA was evaluated using agarose gel electrophoresis and the λ-Hind III digest DNA Marker (Code:D3403A, TaKaRa, China). For each sample, approximately 3 μg DNA with a concentration of 100 ng/μl was obtained.

### Genome sequencing and assembly

Whole-genome sequencing was performed using the Illumina HiSeq 2000 by generating paired-end and mate-pair libraries with an average insert size of 180 bp, 380 bp, 2 kbp and 6 kbp. The length of reads for each library was 100 bp. Duplicate paired reads were filtered out from each library with FastUniq v1.1 [28], and reads that were contaminated by Illumina adapter were removed with the cutadapt tool [29]. Subsequently, reads with ~370×/~330×, ~100×, ~100× and ~100× coverage from each library, respectively, were used to perform the assembly. ALLPATHS-LG Release 42411 [30] was applied to assemble the genomes, which begins by correcting sequencing errors. The GapCloser version 1.12 [31] program was used on the resulting scaffolds to close gaps. After that, ICORN [32] was used to perform corrections on the assembly. Finally, six remaining gaps were completely closed by additional PCR experiments. More details are shown in Additional file 2.

### Genome annotation

As the GM4792 strains are very closed to the strain MG1655, the annotations of GM4792 strains were firstly transferred from MG1655 using RATT [33]. And then, *de novo* annotation was performed on both those regions with imperfectly transferred annotations and the insertions with respect to the stain MG1655. tRNA and rRNA were identified using tRNAscan-SE v1.3.1 [34] and RNAmmer v1.2 [35], respectively. Coding sequences (CDSs) were identified using Prodigal v2.5 [36]. CDSs were translated and analyzed using the NCBI nonredundant database, UniProt (released 2012-10) [37], InterPro v40 [38], TIGRFAMs [39], Pfam [40], and COG [41] databases for functional annotation. Genes with signal peptides and transmembrane helices were predicted with TMHMM v2.0 [42] and SignalP v4.0 [43], respectively. Clustered regularly interspaced short palindromic repeats (CRISPR) were identified with CRT v1.2 [44]. Transcription factors were identified based on the results of domain identification and the DBD database v2.0 [45]. Gene ontology term assignment was performed using the GO database (released 2013-3-30) [46] and Blast2Go Pipeline v2.5.0 [47]. Metabolic pathways were constructed based on the KEGG database (Release 76.0) [48] and KAAS [49]. The complete sets of input parameters used for each program are shown in Table S7 of Additional file 1.

## Genome properties

**Fig. 2:**
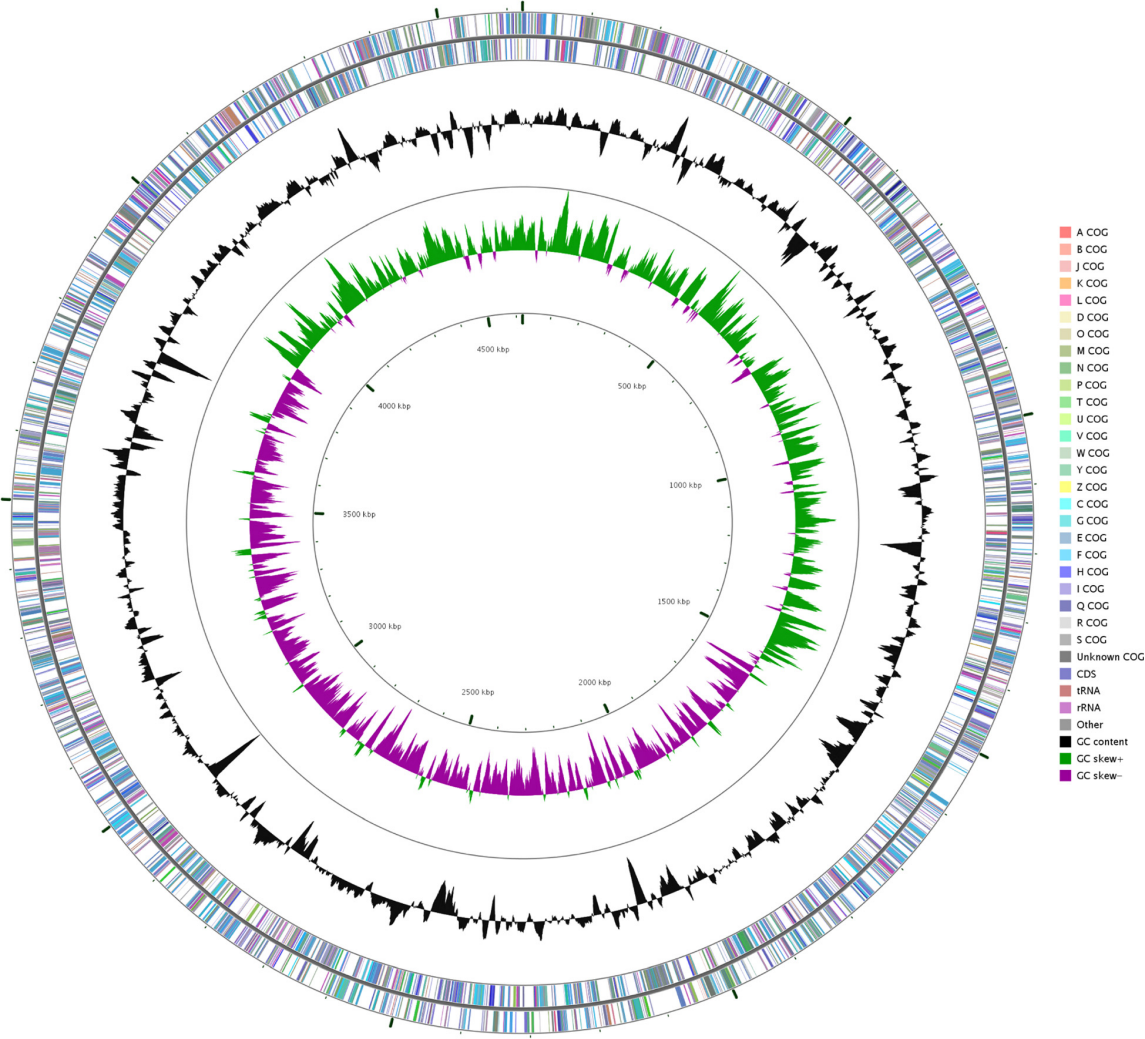
Graphical circular map of the chromosome of *Escherichia coli* K-12 GM4792 Lac^+^. The circles from outside to the inside represent: genes on forward strand (colored by COG categories), genes on reverse strand (colored by COG categories), RNA genes (tRNAs red and rRNAs purple), G + C content (peaks out/inside the circle indicate values higher or lower than the average G + C content, respectively), GC skew (calculated as (G-C)/(G + C), green/purple peaks out/inside the circle indicates values higher or lower than 1, respectively)

**Fig. 3:**
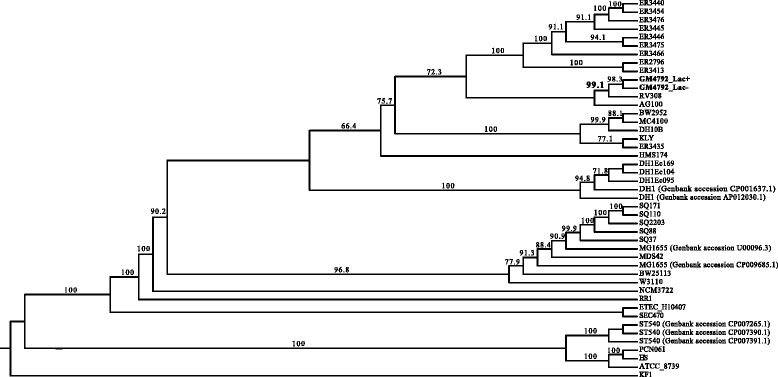
Whole-genome phylogeny highlighting the position of *Escherichia coli* GM4792 relative to the other *E. coli* K-12 strains. Up to 10 October 2015, totally 279 genomes of *Escherichia coli* K-12 strains were released on NCBI. In order to analyze the phylogenetic relationship between GM4792 with other K-12 strains, we downloaded all the genomes of completely assembled and well-annotated K-12 strains. Totally, 44 *Escherichia coli* K-12 strains together with *Escherichia albertii* KF1 as outgroup were used to infer the whole-genome phylogeny using collinear genomic segments [53, 54] (Additional file 1: Table S6). The collinear regions were identified by Sibelia v3.0.6 [55], which can efficiently find LCBs among a large number of microbial genomes without alignment, and then collinear regions shared by all strains were concatenated as supermatrix. The Maximum-likelihood (ML) [56] tree was inferred from the data matrices with FastTree v2.1.8 [57]. Local SH-like support was assessed using Shimodaira-Hasegawa (SH) test with 1000 bootstrap replicates, and the support values are given as names for the internal nodes (values below 60 % have been hidden). *Escherichia albertii* KF1 was used as outgroup to root the tree

GM4792 Lac^+^ genome contains a 4,622,342 bp long chromosome with 50.81 % G + C content. GM4792 Lac^-^ genome has one circular chromosome of 4,621,656 bp with a G + C content of 50.80 %. Totally 4,144 genes were predicted for GM4792 Lac^+^, including 4,061 protein-coding genes and 83 RNA genes (tRNA and rRNA). Similarly, GM4792 Lac^-^ is composed of 4,117 genes (4,043 protein-coding genes and 74 RNA genes). The majority of protein-coding genes, for both GM4792 Lac^+^ and GM4792 Lac^-^, were assigned a putative function (94.64 % and 94.73 %, respectively) and the remaining genes were annotated as hypothetical proteins. The properties and statistics of the two GM4792 strains are summarized in Tables 3 and 4, and the circular maps of the chromosome are shown in Fig. 2 and Figure S3 of the Additional file 1. As GM4792 belongs to K-12 strain, all fully assembled K-12 strains were used for phylogenetic analysis. The other groups may add any further information. All completely assembled and well-annotated K-12 strains were downloaded on 10 October 2015. In order to better characterize the phylogenetic relationships for K-12 strains, *Escherichia albertii* KF1 was included as outgroup. Totally, 45 genomes including 44 *Escherichia coli* K-12 strains were analyzed (Additional file 1: Table S6). According to phylogenetic analysis based on whole-genome sequences, the two GM4792 strains cluster together and are next to *E. coli* RV308 with a high support value (Fig. 3), a similar pattern also supported using a concatenation of single copy protein sequences (Additional file 1: Figure S2).

**Table 3:** Genome statistics

Attribute	Value^b^	% of Total^a,b^	Value^c^	% of Total^a,c^
Genome size (bp)	4,622,342	100.00	4,621,656	100.00
DNA coding (bp)	3,888,159	84.12	3,873,721	83.82
DNA G + C (bp)	2,348,605	50.81	2,348,022	50.80
DNA scaffolds	1		1	0.00
Total genes	4,144	100.00	4,117	100.00
Protein coding genes	4,061	98.00	4,043	98.20
RNA genes	83	2.00	74	1.80
Pseudo genes	0	0.00	0	0.00
Genes in internal clusters	2,036	49.13	2,027	49.23
Genes with function prediction	3,922	94.64	3,900	94.73
Genes assigned to COGs	3,592	88.45	3,580	88.55
Genes with Pfam domains	3,838	92.62	3,818	92.74
Genes with signal peptides	410	9.89	408	9.91
Genes with transmembrane helices	1,058	25.53	1,048	25.46
CRISPR repeats	2		2	

^a^The total based on either the size of the genome in base pairs or the total number of genes in the annotated genome

^b^The genome statistics for GM4792 Lac^+^

^c^The genome statistics for GM4792 Lac^-^

**Table 4:** Number of genes associated with general COG functional categories

Code	Value^b^	% age^a,b^	Value^c^	% age^a,c^	Description
J	258	6.35	259	6.41	Translation, ribosomal structure and biogenesis
A	2	0.05	2	0.05	RNA processing and modification
K	335	8.25	337	8.34	Transcription
L	160	3.94	160	3.96	Replication, recombination and repair
B	0	0.00	0	0.00	Chromatin structure and dynamics
D	50	1.23	50	1.24	Cell cycle control, cell division, chromosome partitioning
Y	0	0.00	0	0.00	Nuclear structure
V	107	2.63	107	2.65	Defense mechanisms
T	251	6.18	250	6.18	Signal transduction mechanisms
M	286	7.04	285	7.05	Cell wall/membrane/envelope biogenesis
N	116	2.86	114	2.82	Cell motility
Z	0	0.00	0	0.00	Cytoskeleton
W	38	0.94	36	0.89	Extracellular structures
U	63	1.55	61	1.51	Intracellular trafficking, secretion, and vesicular transport
O	171	4.21	170	4.20	Posttranslational modification, protein turnover, chaperones
X	31	0.76	31	0.77	Mobilome: prophages, transposons
C	317	7.81	316	7.82	Energy production and conversion
G	437	10.76	438	10.83	Carbohydrate transport and metabolism
E	397	9.78	397	9.82	Amino acid transport and metabolism
F	108	2.66	108	2.67	Nucleotide transport and metabolism
H	189	4.65	188	4.65	Coenzyme transport and metabolism
I	133	3.28	133	3.29	Lipid transport and metabolism
P	263	6.48	260	6.43	Inorganic ion transport and metabolism
Q	77	1.90	77	1.90	Secondary metabolites biosynthesis, transport and catabolism
R	319	7.86	321	7.94	General function prediction only
S	216	5.32	210	5.19	Function unknown
-	469	11.55	463	11.45	Not in COGs

^a^The total is based on the total number of protein coding genes in the genome

^b^The genome statistics for GM4792 Lac^+^

^c^The genome statistics for GM4792 Lac^-^

## Insights from the genome sequence

The paired-end reads with an insert size of 380 bp of Lac^+^ and the scaffolds of Lac^-^ were analyzed using the *breseq* pipeline v0.20 [23] to identify mutations based on read alignments. Six types of variants, including single-base substitution, multiple-base substitution, insertion, deletion, mobile element insertion, and sequence amplification, could be identified. All mutations containing a variant within the adjacent 20 base pairs were removed. Then, mutations that persisted when mapping the reads of Lac^-^ to the genome of Lac^-^ were removed. All of the retained mutations were manually reviewed using the graphical output of the mapping results. After filtering, only two significant variants were left: one 1-bp deletion in *lacI* and one synonymous SNP outside of the *lac* operon (Additional file 1: Table S1). We performed a multiple sequence alignment of the three DNA segments containing the *lacI* and *lac* operons from the MG1655, Lac^-^ and Lac^+^ strains using the CLUSTALW program [50]. We detected a 212-bp deletion, which consisted of the last 16 bp of *lacI*, all of the *lac* promoter and operator, and the first 74 bp of *lacZ*, in both the Lac^-^ and Lac^+^ genomes compared to MG1655. In the Lac^-^ strain, an insertion of a C at bp 961 generates a stop codon at bp 1281. Lacking the promoter and operator, the *lac* operon cannot be transcribed. Therefore, the Lac^-^ strain could not utilize lactose. In Lac^+^, the reverse occurred: a 1-bp deletion in this region. The frameshift mutation 1-bp deletion in *lacI* led to the loss of the stop codon, and thus, *lacI* was fused to the *lac* operon, and consequently, the fused protein was transcribed via the *lacI* promoter (Additional file 1: Figure S4). Thus, GM4792 Lac^+^ could catabolize lactose. This transition is in agreement with previous studies [5, 18, 51]. In addition, the GM4792 strains were compared to MG1655 on the whole-genome scale with Mauve version snapshot_2015-02-25 [52]. For GM4792 Lac^+^, 450 SNPs and 112 indels were identified compared to the MG1655. As to GM4792 Lac^-^, there were totally 441 SNPs and 109 indels compared to the MG1655. More details are shown in Additional file 1: Tables S2–S5.

Phenotypic analysis revealed that the lactose marker was neutral under the conditions used in our studies of experimental evolution of *E. coli* in a nitrogen-limited environment; the ratio of fitness between GM4792 Lac^-^ and GM4792 Lac^+^ was 1.00 (0.994 ~ 1.036, 95 % confidence interval) [22]. Therefore, at both the genotypic and phenotypic levels, these two strains differ only by their ability to utilize lactose, indicating that GM4792 Lac^+^ and GM4792 Lac^-^ are a good system for studies of population evolution and adaption.

## Conclusions

This study presents two closely related genomes, *E. coli* lab strains K-12 GM4792 Lac^+^ and GM4792 Lac^-^, which lay a solid foundation for future variant analysis of evolved lines at the genome scale in evolutionary experiments. A whole-genome comparison of GM4792 Lac^+^ and GM4792 Lac^-^ reveals that the extent of genome-wide differences between GM4792 Lac^+^ and GM4792 Lac^-^ are not significant and are isolated to the loci related to the utilization of lactose. Only two significant variants have been detected. One is a synonymous SNP, and the other is 1-bp deletion that is responsible for lactose utilization in GM4792 Lac^+^. Moreover, phenotypic analysis also showed that GM4792 Lac^+^ and GM4792 Lac^-^ are nearly identical regarding survivability, except for lactose utilization, in a nitrogen-limited environment. All of the results indicate that GM4792 Lac^+^ and GM4792 Lac^-^ with neutral markers are ideal systems for future experimental evolution studies.

## Additional files

Additional file 1:
**Supplementary Tables and Figures.** **Table S1.** Genomic differences between *E. coli* GM4792 Lac^+^ and Lac^-^ detected via reads mapping with *breseq* pipeline. **Table S2.** Structural variations (insertions, deletions) of GM4792 Lac^+^ compared to MG1655 obtained with Mauve. **Table S3.** Structural variations (insertions, deletions) of GM4792 Lac^-^ compared to MG1655 obtained with Mauve. **Table S4.** Nonsynonymous changes in protein sequence of GM4792 Lac^+^ compared to MG1655 obtained with Mauve. **Table S5.** Nonsynonymous changes in protein sequence of GM4792 Lac^-^ compared to MG1655 obtained with Mauve. **Table S6.** 45 complete genomes used in this study. **Table S7.** The complete set of input parameters used for programs. **Figure S1.** Scanning-electron micrograph of strain *E. coli* GM4792 Lac^-^. **Figure S2.** Phylogenetic tree inferred from the supermatrix of proteome sequences under the Maximum-likelihood (ML) criterion. **Figure S3.** Graphical circular map of the chromosome of *Escherichia coli* K-12 GM4792 Lac^-^. **Figure S4.** Mutations related to lactose utilization. (PDF 4042 kb) Additional file 2:
**PCR experiment description.** **Table S8.** The primer sequences for two GM4792 strains. **Figure S5.** The primer design for the large gap (~2,900 bps) in GM4792 Lac^-^. (PDF 124 kb) Additional file 3:
**Title of data: A document containing missing key taxonomic references.** (PDF 96 kb)
