# Supplementary material for: Genome sequences of two closely related strains of Escherichia coli K-12 GM4792
Source: Stand Genomic Sci. 2015 Dec 10;10:125. doi: 10.1186/s40793-015-0114-x (PMC4675052; doi:10.1186/s40793-015-0114-x)
Supplement: Additional file 1: — Supplementary Tables and Figures. Table S1. Genomic differences between E. coli GM4792 Lac+ and Lac- detected via reads mapping with breseq pipeline. Table S2. Structural variations (insertions, deletions) of GM4792 Lac+ compared to MG1655 obtained with Mauve. Table S3. Structural variations (insertions, deletions) of GM4792 Lac- compared to MG1655 obtained with Mauve. Table S4. Nonsynonymous changes in protein sequence of GM4792 Lac+ compared to MG1655 obtained with Mauve. Table S5. Nonsynonymous changes in protein sequence of GM4792 Lac- compared to MG1655 obtained with Mauve. Table S6. 45 complete genomes used in this study. Table S7. The complete set of input parameters used for programs. Figure S1. Scanning-electron micrograph of strain E. coli GM4792 Lac-. Figure S2. Phylogenetic tree inferred from the supermatrix of proteome sequences under the Maximum-likelihood (ML) criterion. Figure S3. Graphical circular map of the chromosome of Escherichia coli K-12 GM4792 Lac-. Figure S4. Mutations related to lactose utilization. (PDF 4042 kb) [file 40793_2015_114_MOESM1_ESM.pdf]

Genome sequences of two closely related strains of *Escherichia coli* K-12 GM4792

Yan-Cong Zhang[1], Yan Zhang[1*], Bi-Ru Zhu[1], Bo-Wen Zhang[1], Chuan Ni[1#], Da-Yong Zhang[1], Ying Huang[2], Erli Pang[1], Kui Lin[1]

[1]State Key Laboratory of Earth Surface Processes and Resource Ecology and MOE Key Laboratory for Biodiversity Science and Ecological Engineering, College of Life Sciences, Beijing Normal University, Beijing 100875, China

[2] State Key Laboratory for Infectious Disease Prevention and Control, and National Institute for Communicable Disease Control and Prevention, Chinese Center for Disease Control and Prevention, Beijing 102206, China

Corresponding author:
Kui Lin
College of Life Sciences, Beijing Normal University, 19 Xinjiekouwai Street, Beijing 100875, China
Email: linkui@bnu.edu.cn
Tel: 86 10 58805045
Fax: 86 10 58807721

[*] Current address: National Laboratory of Biomacromolecules, Institute of Biophysics, Chinese Academy of Sciences, Beijing 100101, China

[#] Current address: The second high school attached to Beijing Normal University, Beijing 100192, China

Figure legends

Figure S1 Scanning-electron micrograph of strain *E. coli* GM4792 Lac$^-$.

Figure S2 Phylogenetic tree inferred from the supermatrix of proteome sequences under the Maximum-likelihood (ML) **criterion.**

Protein-coding sequences from 44 K-12 strains of *E. coli* together with *Escherichia albertii* KF1 as outgroup downloaded from NCBI were phylogenetically analyzed in a complementary analysis using the phylogenomics pipeline as previously described [1]. The concatenated alignments of distinct selections of genes (supermatrix) were generated using NCBI BLAST [2], OrthoMCL v2.0.5 [3], clustal-omega v1.1.0 [4], mafft v 7.164 [5], T-coffee v9.03.r1318 [6], mumsa v1.0 [7] and trimAl v1.4 [8]. The Maximum-likelihood (ML) [9] tree was inferred from the data matrices with FastTree v2.1.8 [10]. Local SH-like support was assessed using Shimodaira-Hasegawa (SH) test with 1000 bootstrap replicates, and the support values are given as names for the internal nodes. The tree was rooted with *Escherichia albertii* KF1 as outgroup.

Figure S3 Graphical circular map of the chromosome of *Escherichia coli* K-12 GM4792 Lac$^-$.

The circles from outside to the inside represent: genes on forward strand (colored by COG categories), genes on reverse strand (colored by COG categories), RNA genes (tRNAs red and rRNAs purple), G+C content (peaks out/inside the circle indicate values higher or lower than the average G+C content, respectively), GC skew (calculated as (G-C)/(G+C), green/purple peaks out/inside the circle indicates values higher or lower than 1, respectively).

Figure S4 Mutations related to lactose utilization.

Numbering is initiated at the start codon of *lacI*. Highlighted in yellow is the insertion of the C at base pair 961, which generates a stop codon at base pair 1281. There exists a 212-bp deletion from base pair 1069 to 1280, consisting of the last 16 bp of *lacI*, all of the *lac* promoter and operator, and the first 74 bp of *lacZ*, in both the Lac$^-$ and Lac$^+$ genomes compared to MG1655.

Tables

Table S1 - Genomic differences between *E. coli* GM4792 Lac$^+$ and Lac$^-$ detected via reads mapping with *breseq* pipeline.

Seq ID	Position	Mutation	Annotation	Gene	Description	Quality Control[a]
GM4792 Lac$^-$	365,105	Δ1 bp	coding (970/1080 nt)	*lacI*	lactose-inducible *lac* operon transcriptional repressor protein	PASS
GM4792 Lac$^-$	737,035	G→T	R95L (CGT→CTT)	*ybfD*	H repeat-associated putative transposase protein	1
GM4792 Lac$^-$	737,041	G→A	C97Y (TGC→TAC)	*ybfD*	H repeat-associated putative transposase protein	1
GM4792 Lac$^-$	737,069	T→C	I106I (ATT→ATC)	*ybfD*	H repeat-associated putative transposase protein	1, 2
GM4792 Lac$^-$	737,086	2 bp→TA	coding (335-336/762 nt)	*ybfD*	H repeat-associated putative transposase protein	1, 2
GM4792 Lac$^-$	737,089	T→A	L113H (CTC→CAC)	*ybfD*	H repeat-associated putative transposase protein	1, 2
GM4792 Lac$^-$	737,096	C→T	H115H (CAC→CAT)	*ybfD*	H repeat-associated putative transposase protein	1, 2
GM4792 Lac$^-$	1,213,584	Δ67 bp	intergenic (-97/-277)	*hlyE/umuD*	hemolysin E protein/DNA polymerase V, subunit D protein	3
GM4792 Lac$^-$	1,239,094	Δ37 bp	intergenic (-219/-364)	*ycgV/U069_c3711*	putative adhesin protein/ trans_membrane protein	2

GM4792 Lac$^-$	1,563,726	C→T	intergenic (-274/+181)	*ydeN/ydeO*	putative Ser-type periplasmic non-arylsulfatase protein/UV-inducible global regulator, EvgA-,GadE-dependent protein	2
GM4792 Lac$^-$	1,613,635	C→T	intergenic (-369/-418)	*U069_c3764/ydfK*	Secretory pathway trans_membrane protein/cold shock protein, Qinprophage protein	3
GM4792 Lac$^-$	1,613,897	G→T	intergenic (-631/-156)	*U069_c3764/ydfK*	Secretory pathway trans_membrane protein/cold shock protein, Qinprophage protein	2
GM4792 Lac$^-$	2,236,095	+GAATAATTTTTCTCTTTTCCA	coding (2/921 nt)	*rihB*	ribonucleoside hydrolase 2 protein	2
GM4792 Lac$^-$	3,363,413	C→T	R170R (CGG →CGA)	*aaeB*	p-hydroxybenzoic acid efflux system component protein	PASS
GM4792 Lac$^-$	3,741,906	A→G	intergenic (-2985/-4393)	*yibF/yibA*	glutathione S-transferase-like protein/HEAT-domain lethality reduction protein putative immunity protein	2

[a]1: Variations that were removed because the distance to the nearest variant was less than 20bp; 2: Variations that were removed because they were also identified when mapping the reads of Lac$^-$ onto the Lac$^-$ reference genome; 3: Variations that were discarded due to manual inspection of the graphical output of the reads mapping; PASS: variants that passed the above three quality control measures.

Table S2 - Structural variations (insertions, deletions) of GM4792 Lac$^+$ compared to MG1655 obtained with Mauve.

MG1655 -specific	Lac$^+$ -specific	Start	Stop	Length (bp)	Description
✓		224,481	224,773	293	rrsH
✓		225,356	226,213	858	alaV;ileV;rrlH
✓		257,908	258,687	780	crl;mobile_element
✓		325,238	325,270	33	ykgH
✓		339,900	339,996	97	repeat_region
✓		349,788	349,884	97	repeat_region
✓		366,230	366,445	216	lacI;lacZ
✓		1,097,164	1,097,529	366	Intergenic
✓		1,196,238	1,211,445	15,208	aaaE;beeE;cohE;croE;icd;icdC;intE;jayE;lit;mcrA;oweE;pinE;stfE;stfP;tfaE;tfaP;xisE;ymfD;ymfE;ymfI;ymfJ;ymfL;ymfM;ymfQ;ymfR
✓		1,269,284	1,269,822	539	ldrB;rdlA
✓		1,299,493	1,300,695	1,203	mobile_element
✓		1,552,016	1,552,197	182	repeat_region
✓		1,870,570	1,870,657	88	yeaI
✓		1,978,493	1,979,272	780	insA;insB1;mobile_element
✓		2,304,458	2,304,800	343	repeat_region
✓		2,558,699	2,565,492	6,794	cryptic prophage Eut/CPZ-55;eutA;intZ;yffL;yffM;yffN;yffO;yffP;yffQ;yffR;yffS
✓		2,991,937	2,992,060	124	Intergenic
✓		3,269,918	3,270,034	117	Intergenic
✓		3,392,194	3,392,288	95	repeat_region
✓		3,423,506	3,423,754	249	rrfD;rrfF;thrV
✓		3,799,065	3,799,257	193	waaU
✓		4,037,132	4,037,359	228	alaT;ileT
✓		4,094,314	4,094,501	188	repeat_region
✓		4,209,146	4,209,735	590	rrsE
✓		4,282,295	4,282,388	94	yjcF
✓		4,295,929	4,296,382	454	repeat_region
✓		4,326,015	4,326,318	304	repeat_region

✓	66,773	66,860	88	repeat_region
✓	224,566	226,206	1,641	RBS;alaV;rrlH
✓	735,174	735,556	383	GIS
✓	1,852,261	1,852,287	27	yeaI
✓	2,156,375	2,157,153	779	RBS;mobile_element
✓	2,328,518	2,328,615	98	repeat_region
✓	2,449,679	2,450,942	1,264	Intergenic
✓	2,967,807	2,967,843	37	Intergenic
✓	2,969,479	2,969,525	47	Intergenic
✓	3,326,258	3,327,036	779	Intergenic
✓	3,406,137	3,407,400	1,264	Intergenic
✓	3,743,499	3,747,254	3,756	Intergenic
✓	3,780,233	3,780,279	47	Intergenic
✓	3,869,891	3,870,670	780	Intergenic
✓	4,018,935	4,019,069	135	alaT

Table S3 - Structural variations (insertions, deletions) of GM4792 Lac$^-$ compared to MG1655 obtained with Mauve.

MG1655 -specific	Lac$^-$ -specific	Start	Stop	Length (bp)	Description
✓		66,602	66,690	89	repeat_region
✓		257,908	258,687	780	crl;mobile_element
✓		339,757	339,853	97	repeat_region
✓		339,928	340,024	97	repeat_region
✓		349,779	349,968	190	repeat_region
✓		366,230	366,445	216	lacI;lacZ
✓		375,125	375,330	206	repeat_region
✓		636,843	636,875	33	ybdO
✓		1,097,216	1,097,581	366	serX
✓		1,196,238	1,211,445	15,208	aaaE;beeE;cohE;croE;icd;icdC;intE;jayE;lit;mcrA;oweE;pinE;stfE;stfP;tfaE;tfaP;xisE;ymfD;ymfE;ymfI;ymfJ;ymfL;ymfM;ymfQ;ymfR
✓		1,269,361	1,270,434	1,074	ldrB;ldrC;rdlA;rdlB;rdlC
✓		1,299,493	1,300,695	1,203	mobile_element
✓		1,978,493	1,979,272	780	insA;insB1;mobile_element
✓		2,052,568	2,052,618	51	yeeL
✓		2,255,159	2,255,183	25	rihB
✓		2,304,566	2,305,021	456	repeat_region
✓		2,558,699	2,565,492	6,794	cryptic prophage Eut/CPZ-55;eutA;intZ;yffL;yffM;yffN;yffO;yffP;yffQ;yffR;yffS
✓		2,729,445	2,729,550	106	Intergenic
✓		2,933,958	2,933,995	38	Intergenic
✓		3,269,947	3,270,063	117	Intergenic
✓		3,426,934	3,427,161	228	alaU;ileU
✓		3,765,072	3,765,832	761	rhsA
✓		3,943,410	3,943,545	136	gltU
✓		4,094,557	4,094,652	96	repeat_region
✓		4,153,358	4,153,484	127	repeat_region
✓		4,296,042	4,296,382	341	repeat_region
✓		4,326,003	4,326,306	304	repeat_region

✓	694,753	695,091	339	terminator
✓	1,213,569	1,213,638	70	Intergenic
✓	1,239,074	1,239,113	40	Intergenic
✓	1,563,671	1,563,726	56	Intergenic
✓	2,155,679	2,156,457	779	RBS;mobile_element
✓	2,448,754	2,450,017	1,264	Intergenic
✓	2,759,592	2,759,619	28	Intergenic
✓	2,968,158	2,968,389	232	ygeH
✓	3,325,513	3,326,291	779	Intergenic
✓	3,402,677	3,402,811	135	alaU
✓	3,405,636	3,406,899	1,264	Intergenic
✓	3,628,065	3,628,120	56	Intergenic
✓	3,742,298	3,746,053	3,756	RBS
✓	3,868,831	3,869,610	780	Intergenic
✓	3,924,150	3,924,376	227	Intergenic

Table S4 - Nonsynonymous changes in protein sequence of GM4792 Lac$^+$ compared to MG1655 obtained with Mauve.

Seq ID	Position	Mutation	Amino acid change	Gene	Description
NC_000913.3	70,659	G->A	W->*	araC	ara regulon transcriptional activator; autorepressor
NC_000913.3	100,946	T->C	M->T	murC	UDP-N-acetylmuramate:L-alanine ligase
NC_000913.3	480,295	G->A	P->L	hha	modulator of gene expression, with H-NS
NC_000913.3	480,296	G->A	P->S	hha	modulator of gene expression, with H-NS
NC_000913.3	574,897	C->T	R->K	insH1	IS5 transposase and trans-activator
NC_000913.3	700,038	G->A	H->Y	umpH	UMP phosphatase
NC_000913.3	732,286	A->G	T->A	rhsC	Rhs family putative polymorphic toxin
NC_000913.3	732,340	G->A	A->T	rhsC	Rhs family putative polymorphic toxin
NC_000913.3	732,533	C->A	A->E	rhsC	Rhs family putative polymorphic toxin
NC_000913.3	809,217	C->A	W->L	bioA	7,8-diaminopelargonic acid synthase, PLP-dependent
NC_000913.3	809,218	A->G	W->R	bioA	7,8-diaminopelargonic acid synthase, PLP-dependent
NC_000913.3	968,837	G->A	D->N	lpxK	lipid A 4'kinase
NC_000913.3	1,061,935	G->A	S->N	torD	TorA-maturation chaperone
NC_000913.3	1,069,743	C->T	A->T	rutG	pyrimidine permease
NC_000913.3	1,111,727	C->T	P->S	opgH	membrane glycosyltransferase; nutrient-dependent cell size regulator, FtsZ assembly antagonist
NC_000913.3	1,111,728	C->T	P->L	opgH	membrane glycosyltransferase; nutrient-dependent cell size regulator, FtsZ assembly antagonist
NC_000913.3	1,169,836	A->G	L->P	ldtC	L,D-transpeptidase linking

					Lpp to murein
NC_000913.3	1,269,191	T->C	S->G	ldrA	toxic polypeptide, small
NC_000913.3	1,269,250	A->G	I->T	ldrA	toxic polypeptide, small
NC_000913.3	1,301,992	A->T	N->Y	oppA	oligopeptide transporter subunit
NC_000913.3	1,306,736	T->G	S->A	oppF	oligopeptide transporter subunit
NC_000913.3	1,337,394	A->G	S->G	acnA	aconitate hydratase 1
NC_000913.3	1,358,859	T->C	Y->C	puuP	putrescine importer
NC_000913.3	1,432,657	A->G	N->D	tfaR	Rac prophage; putative tail fiber assembly protein
NC_000913.3	1,433,199	A->G	V->A	pinR	Rac prophage; putative site-specific recombinase
NC_000913.3	1,433,667	C->T	R->Q	pinR	Rac prophage; putative site-specific recombinase
NC_000913.3	1,539,947	C->G	A->P	narZ	nitrate reductase 2 (NRZ), alpha subunit
NC_000913.3	1,633,223	A->C	K->T	ydfK	cold shock protein, function unknown, Qin prophage
NC_000913.3	1,633,629	A->G	Q->R	pinQ	Qin prophage; putative site-specific recombinase
NC_000913.3	1,634,097	C->T	A->V	pinQ	Qin prophage; putative site-specific recombinase
NC_000913.3	1,634,639	C->T	D->N	tfaQ	Qin prophage; putative tail fiber assembly protein
NC_000913.3	1,643,679	A->T	L->Q	ydfU	Qin prophage; uncharacterized protein
NC_000913.3	1,652,331	T->C	F->L	intQ	
NC_000913.3	1,870,565	T->C	F->L	yeaI	putative membrane-anchored diguanylate cyclase
NC_000913.3	1,870,566	T->G	F->C	yeaI	putative membrane-anchored diguanylate cyclase
NC_000913.3	1,894,839	T->C	L->P	pabB	aminodeoxychorismate synthase, subunit I
NC_000913.3	2,040,433	C->A	A->D	yedY	membrane-anchored, periplasmic TMAO, DMSO reductase

NC_000913.3	2,587,112	C->T	A->V	narQ	sensory histidine kinase in two-component regulatory system with NarP (NarL)
NC_000913.3	2,867,454	T->A	Q->L	rpoS	RNA polymerase, sigma S (sigma 38) factor
NC_000913.3	2,867,455	G->A	Q->*	rpoS	RNA polymerase, sigma S (sigma 38) factor
NC_000913.3	2,939,297	G->T	G->V	fucU	L-fucose mutarotase
NC_000913.3	3,152,494	G->T	A->S	metC	cystathionine beta-lyase, PLP-dependent
NC_000913.3	3,329,743	G->T	D->Y	dacB	D-alanyl-D-alanine carboxypeptidase
NC_000913.3	3,388,041	T->G	T->P	aaeB	p-hydroxybenzoic acid efflux system component
NC_000913.3	3,560,443	C->G	G->A	glpR	
NC_000913.3	3,725,176	T->G	E->A	glyQ	glycine tRNA synthetase, alpha subunit
NC_000913.3	3,763,974	A->G	I->V	rhsA	Rhs family protein, putative polymorphic toxin; putative polysaccharide synthesis/export protein; putative neighboring cell growth inhibitor
NC_000913.3	3,764,886	G->A	A->T	rhsA	Rhs family protein, putative polymorphic toxin; putative polysaccharide synthesis/export protein; putative neighboring cell growth inhibitor
NC_000913.3	3,764,940	A->G	T->A	rhsA	Rhs family protein, putative polymorphic toxin; putative polysaccharide synthesis/export protein; putative neighboring cell growth inhibitor
NC_000913.3	3,948,215	G->T	E->*	yifE	UPF0438 family protein
NC_000913.3	4,133,881	G->A	C->Y	katG	catalase-peroxidase HPI, heme b-containing
NC_000913.3	4,325,644	C->T	S->N	yjdN	metalloprotein superfamily protein

NC_000913.3	4,473,900	C->T	G->R	bdcA	c-di-GMP-binding biofilm dispersal mediator protein
NC_000913.3	4,636,925	G->C	R->P	creC	sensory histidine kinase in two-component regulatory system with CreB or PhoB, regulator of the CreBC regulon

Table S5 - Nonsynonymous changes in protein sequence of GM4792 Lac⁻ compared to MG1655 obtained with Mauve.

Seq ID	Position	Mutation	Amino acid change	Gene	Description
NC_000913.3	70,659	G->A	W->*	araC	ara regulon transcriptional activator; autorepressor
NC_000913.3	100,946	T->C	M->T	murC	UDP-N-acetylmuramate:L-alanine ligase
NC_000913.3	271,383	G->C	V->L	insI1	IS30 transposase
NC_000913.3	290,958	C->A	R->S	insB1	IS1 transposase B
NC_000913.3	291,098	A->G	F->L	insB1	IS1 transposase B
NC_000913.3	291,101	G->A	P->S	insB1	IS1 transposase B
NC_000913.3	291,104	G->T	P->T	insB1	IS1 transposase B
NC_000913.3	291,104	G->T	F->L	insA	IS1 repressor TnpA
NC_000913.3	291,140	G->T	R->S	insB1	IS1 transposase B
NC_000913.3	381,681	G->A	A->T	insC1	IS2 repressor TnpA
NC_000913.3	381,681	G->A	S->N	insD1	IS2 transposase TnpB
NC_000913.3	381,682	C->A	A->E	insC1	IS2 repressor TnpA
NC_000913.3	381,682	C->A	S->R	insD1	IS2 transposase TnpB
NC_000913.3	480,295	G->A	P->L	hha	modulator of gene expression, with H-NS
NC_000913.3	480,296	G->A	P->S	hha	modulator of gene expression, with H-NS
NC_000913.3	700,038	G->A	H->Y	umpH	UMP phosphatase
NC_000913.3	732,286	A->G	T->A	rhsC	Rhs family putative polymorphic toxin
NC_000913.3	732,340	G->A	A->T	rhsC	Rhs family putative polymorphic toxin
NC_000913.3	732,533	C->A	A->E	rhsC	Rhs family putative polymorphic toxin
NC_000913.3	732,707	C->G	T->S	rhsC	Rhs family putative polymorphic toxin
NC_000913.3	732,811	A->G	T->A	rhsC	Rhs family putative polymorphic toxin
NC_000913.3	738,255	C->T	P->L	ybfD	H repeat-associated putative transposase
NC_000913.3	738,335	T->A	C->S	ybfD	H repeat-associated putative transposase
NC_000913.3	738,356	A->T	S->C	ybfD	H repeat-associated

					putative transposase
NC_000913.3	738,375	T->G	L->R	ybfD	H repeat-associated putative transposase
NC_000913.3	738,381	A->G	Y->C	ybfD	H repeat-associated putative transposase
NC_000913.3	738,426	T->C	I->T	ybfD	H repeat-associated putative transposase
NC_000913.3	738,427	A->G	I->M	ybfD	H repeat-associated putative transposase
NC_000913.3	738,429	A->T	H->L	ybfD	H repeat-associated putative transposase
NC_000913.3	809,217	C->A	W->L	bioA	7,8-diaminopelargonic acid synthase, PLP-dependent
NC_000913.3	809,218	A->G	W->R	bioA	7,8-diaminopelargonic acid synthase, PLP-dependent
NC_000913.3	968,837	G->A	D->N	lpxK	lipid A 4'kinase
NC_000913.3	1,061,935	G->A	S->N	torD	TorA-maturation chaperone
NC_000913.3	1,069,743	C->T	A->T	rutG	pyrimidine permease
NC_000913.3	1,111,727	C->T	P->S	opgH	membrane glycosyltransferase; nutrient-dependent cell size regulator, FtsZ assembly antagonist
NC_000913.3	1,111,728	C->T	P->L	opgH	membrane glycosyltransferase; nutrient-dependent cell size regulator, FtsZ assembly antagonist
NC_000913.3	1,169,836	A->G	L->P	ldtC	L,D-transpeptidase linking Lpp to murein
NC_000913.3	1,301,992	A->T	N->Y	oppA	oligopeptide transporter subunit
NC_000913.3	1,306,736	T->G	S->A	oppF	oligopeptide transporter subunit
NC_000913.3	1,337,394	A->G	S->G	acnA	aconitate hydratase 1
NC_000913.3	1,358,859	T->C	Y->C	puuP	putrescine importer
NC_000913.3	1,539,947	C->G	A->P	narZ	nitrate reductase 2 (NRZ), alpha subunit
NC_000913.3	1,643,679	A->T	L->Q	ydfU	Qin prophage; uncharacterized protein

NC_000913.3	1,652,331	T->C	F->L	intQ	
NC_000913.3	1,894,839	T->C	L->P	pabB	aminodeoxychorismate synthase, subunit I
NC_000913.3	2,040,433	C->A	A->D	yedY	membrane-anchored, periplasmic TMAO, DMSO reductase
NC_000913.3	2,066,465	T->C	K->R	insH1	IS5 transposase and trans-activator
NC_000913.3	2,066,996	C->A	S->I	insH1	IS5 transposase and trans-activator
NC_000913.3	2,587,112	C->T	A->V	narQ	sensory histidine kinase in two-component regulatory system with NarP (NarL)
NC_000913.3	2,867,454	T->A	Q->L	rpoS	RNA polymerase, sigma S (sigma 38) factor
NC_000913.3	2,867,455	G->A	Q->*	rpoS	RNA polymerase, sigma S (sigma 38) factor
NC_000913.3	2,939,297	G->T	G->V	fucU	L-fucose mutarotase
NC_000913.3	3,152,494	G->T	A->S	metC	cystathionine beta-lyase, PLP-dependent
NC_000913.3	3,186,583	G->A	R->H	insD1	IS2 transposase TnpB
NC_000913.3	3,329,743	G->T	D->Y	dacB	D-alanyl-D-alanine carboxypeptidase
NC_000913.3	3,388,041	T->G	T->P	aaeB	p-hydroxybenzoic acid efflux system component
NC_000913.3	3,560,443	C->G	G->A	glpR	
NC_000913.3	3,620,162	T->C	V->A	rhsB	Rhs family putative polymorphic toxin, putative neighboring cell growth inhibitor
NC_000913.3	3,620,179	G->A	G->S	rhsB	Rhs family putative polymorphic toxin, putative neighboring cell growth inhibitor
NC_000913.3	3,620,180	G->A	G->D	rhsB	Rhs family putative polymorphic toxin, putative neighboring cell growth inhibitor
NC_000913.3	3,620,371	G->A	D->N	rhsB	Rhs family putative polymorphic toxin, putative

					neighboring cell growth inhibitor
NC_000913.3	3,620,644	T->A	L->M	rhsB	Rhs family putative polymorphic toxin, putative neighboring cell growth inhibitor
NC_000913.3	3,620,666	T->G	L->W	rhsB	Rhs family putative polymorphic toxin, putative neighboring cell growth inhibitor
NC_000913.3	3,622,316	G->C	S->T	rhsB	Rhs family putative polymorphic toxin, putative neighboring cell growth inhibitor
NC_000913.3	3,622,670	C->A	T->K	rhsB	Rhs family putative polymorphic toxin, putative neighboring cell growth inhibitor
NC_000913.3	3,622,678	G->A	A->T	rhsB	Rhs family putative polymorphic toxin, putative neighboring cell growth inhibitor
NC_000913.3	3,622,685	C->A	A->E	rhsB	Rhs family putative polymorphic toxin, putative neighboring cell growth inhibitor
NC_000913.3	3,725,176	T->G	E->A	glyQ	glycine tRNA synthetase, alpha subunit
NC_000913.3	3,764,886	G->A	A->T	rhsA	Rhs family protein, putative polymorphic toxin; putative polysaccharide synthesis/export protein; putative neighboring cell growth inhibitor
NC_000913.3	3,948,215	G->T	E->*	yifE	UPF0438 family protein
NC_000913.3	4,133,881	G->A	C->Y	katG	catalase-peroxidase HPI, heme b-containing
NC_000913.3	4,325,644	C->T	S->N	yjdN	metalloprotein superfamily protein
NC_000913.3	4,473,900	C->T	G->R	bdcA	c-di-GMP-binding biofilm

					dispersal mediator protein
NC_000913.3	4,498,602	G->A	A->T	insC1	IS2 repressor TnpA
NC_000913.3	4,498,602	G->A	S->N	insD1	IS2 transposase TnpB
NC_000913.3	4,498,603	C->A	A->E	insC1	IS2 repressor TnpA
NC_000913.3	4,498,603	C->A	S->R	insD1	IS2 transposase TnpB
NC_000913.3	4,507,837	G->C	L->V	insI1	IS30 transposase
NC_000913.3	4,636,925	G->C	R->P	creC	sensory histidine kinase in two-component regulatory system with CreB or PhoB, regulator of the CreBC regulon

Table S6 - 45 complete genomes used in this study.

Strain	Size (Mb)	Accession number
Escherichia coli		
K-12 substr. MG1655	4.64165	U00096.3
K-12 substr. W3110	4.64633	AP009048.1
HS	4.64354	CP000802.1
ATCC 8739	4.74622	CP000946.1
K-12 substr. DH10B	4.68614	CP000948.1
K-12 substr. BW2952	4.57816	CP001396.1
DH1	4.63071	CP001637.1
ETEC H10407	5.32589	FN649414.1
DH1	4.62143	AP012030.1
K-12 substr. MDS42	3.9762	AP012306.1
K-12 substr. MC4100	4.52725	HG738867.1
ST540	4.75863	CP007265.1
ST540	4.80798	CP007390.1
ST540	4.87568	CP007391.1
KLY	4.71854	CP008801.1
K-12 substr. BW25113	4.63147	CP009273.1
ER2796	4.55866	CP009644.1
ER3413	4.55866	CP009789.1
K-12 substr. MG1655	4.63683	CP009685.1
K-12 substr. RV308	4.58562	LM995446.1
K-12 substr. HMS174	4.58486	LM993812.1
ER3454	4.61973	CP010438.1
ER3440	4.60454	CP010439.1
ER3476	4.62116	CP010440.1
ER3445	4.60763	CP010441.1
ER3466	4.66043	CP010442.1
ER3446	4.60686	CP010443.1
ER3475	4.60963	CP010444.1
ER3435	4.68209	CP010445.1
K-12 substr. AG100	4.63813	LN832404.1
SEC470	5.15343	CP007594.1
SQ37	4.63346	CP011320.1
SQ88	4.61088	CP011321.1
SQ110	4.60513	CP011322.1
SQ171	4.5997	CP011323.1
SQ2203	4.6053	CP011324.1
PCN061	4.90182	CP006636.1
NCM3722	4.74559	CP011495.1
DH1Ec095	4.61422	CP012125.1
DH1Ec104	4.61236	CP012126.1
DH1Ec169	4.60952	CP012127.1
RR1	4.58729	CP011113.1
GM4792 Lac$^-$	4.62166	CP011343.1
GM4792 Lac$^+$	4.62234	CP011342.1
Escherichia albertii		
KF1	4.70188	CP007025.1

Table S7 - The complete set of input parameters used for programs.

Programs	Parameters
FastUniq v1.1	-i <filelist.txt> -o <read_left.fq> -p <read_right.fq>
ALLPATHS-LG Release 42411	default
GapCloser v1.12	-l 100 -p 25 -a <input.scaf.file > -b <input.lib.conf><br>-o <output.scaf.gc.file>
ICORN v0.97	<iteration start, 1> <iteration stop, 5>
RATT	<Directory with embl-files> <genome.fa> <Resultname> Strain
tRNAscan-SE v1.3.1	-o <tRNAscan.results> -f <tRNA.structure><br>-m <tRNA.statistics> -H <genome.fa>
RNAmmer v1.2	-S bac -gff <result.gff> -m tsu,ssu,lsu
Prodigal v2.5	-t train -a <pro.fasta> -c -d <cds.fasta> -f gff -i "refseq"<br>-o <my.gene>
TMHMM v2.0	-short "seq"
SignalP v4.0	-f short -l log -n gff -t gram- "seq"
CRT v1.2	crt "seq" "seq.out"
Blast2Go Pipeline v2.5.0	-in <results.xml> -out <go.txt> -prop b2gPipe.properties -annot
KAAS v2.0	default
Mauve snapshot_2015-02-25	default
CLUSTALW v 2.1	default
FastTree v2.1.8	default
Sibelia version 3.0.6	-s loose -q -g -v -t tmp --gff

References

1. Roquet C, Thuiller W, Lavergne S. **Building megaphylogenies for macroecology: taking up the challenge**. Ecography. 2013;**36(1)**:13-26.
2. Altschul SF, Gish, W., Miller, W., Myers, E. W., & Lipman, D. J. **Basic local alignment search tool.** Journal of Molecular Biology. 1990;**215(3)**:403-10.
3. Li L, Stoeckert CJ, Roos DS. **OrthoMCL: Identification of ortholog groups for eukaryotic genomes**. Genome Research. 2003;**13(9)**:2178-89.
4. Sievers F, Wilm A, Dineen D, Gibson TJ, Karplus K, Li W et al. **Fast, scalable generation of high-quality protein multiple sequence alignments using Clustal Omega**. Molecular Systems Biology. 2011;**7**.
5. Katoh K, Kuma K, Toh H, Miyata T. **MAFFT version 5: improvement in accuracy of multiple sequence alignment**. Nucleic Acids Research. 2005;**33(2)**:511-8.
6. Notredame C, Higgins DG, Heringa J. **T-Coffee: A novel method for fast and accurate multiple sequence alignment**. Journal of Molecular Biology. 2000;**302(1)**:205-17.
7. Lassmann T, Sonnhammer ELL. **Kalign, Kalignvu and Mumsa: web servers for multiple sequence alignment**. Nucleic Acids Research. 2006;**34**:W596-W9.
8. Capella-Gutierrez S, Silla-Martinez JM, Gabaldon T. **trimAl: a tool for automated alignment trimming in large-scale phylogenetic analyses**. Bioinformatics. 2009;**25(15)**:1972-3.
9. Felsenstein J. **Evolutionary trees from DNA sequences: a maximum likelihood approach**. Journal of Molecular Evolution. 1981;**17(6)**:368-76.
10. Price MN, Dehal PS, Arkin AP. **FastTree 2 – approximately maximum-likelihood trees for large alignments**. Plos One. 2010;**5(3)**:e9490.
11. Saitou N, Nei M. **The neighbor-joining method: a new method for reconstructing phylogenetic trees**. Molecular biology and evolution. 1987;**4(4)**:406-25.
12. Plotree D, Plotgram D. **PHYLIP-phylogeny inference package (version 3.2)**. cladistics. 1989;**5**:163-6.
13. Angiuoli SV, Salzberg SL. **Mugsy: fast multiple alignment of closely related whole genomes**. Bioinformatics. 2011;**27(3)**:334-42.

Figures

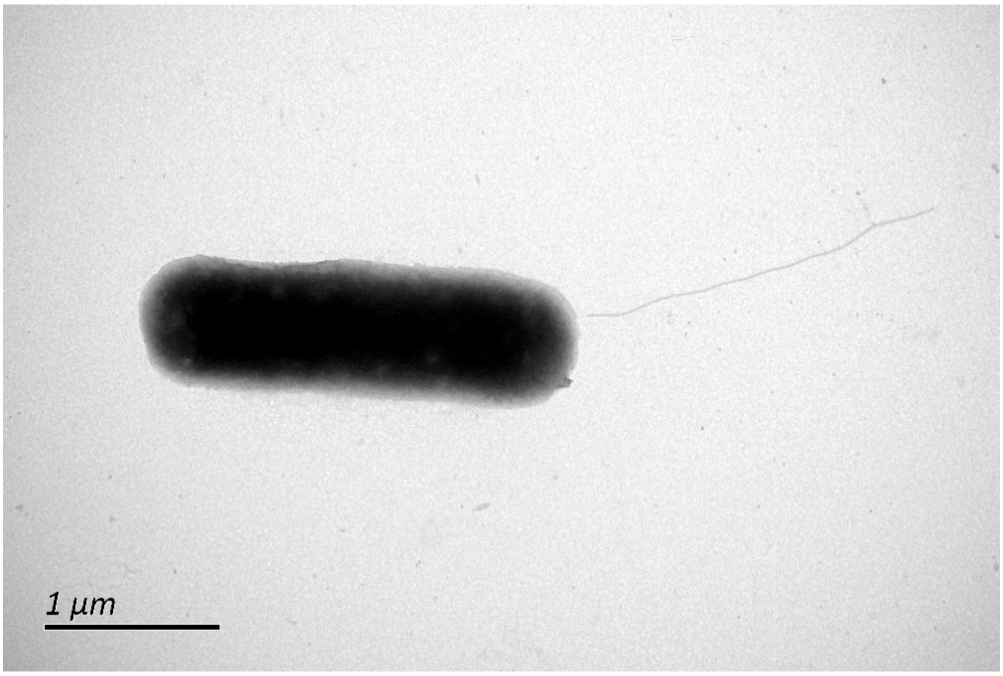

Figure S1

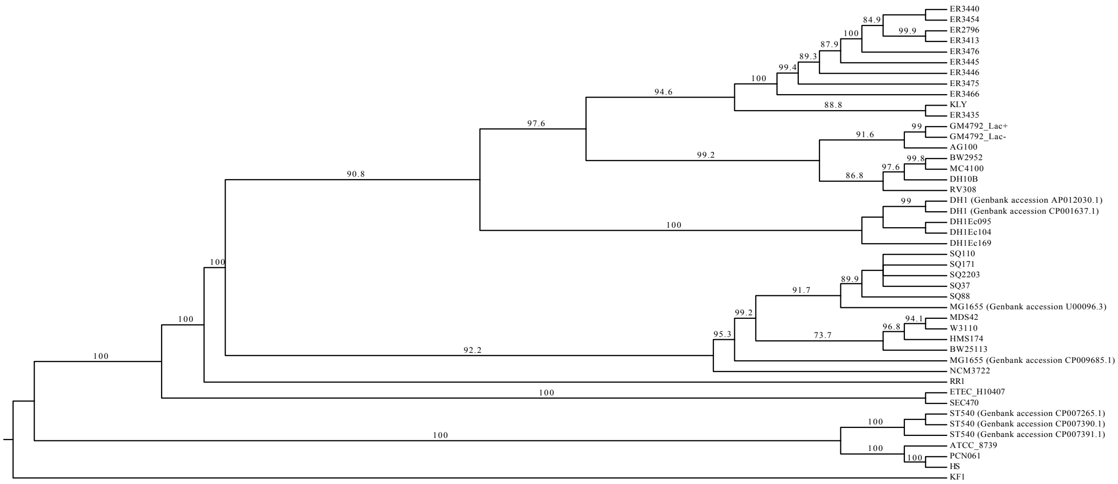
ER3440
ER3454
ER2796
ER3413
ER3476
ER3445
ER3446
ER3475
ER3466
KLY
ER3435
GM4792_Lac+
GM4792_Lac-
AG100
BW2952
MC4100
DH10B
RV308
DH1 (Genbank accession AP012030.1)
DH1 (Genbank accession CP001637.1)
DH1Ec095
DH1Ec104
DH1Ec169
SQ110
SQ171
SQ2203
SQ37
SQ88
MG1655 (Genbank accession U00096.3)
MDS42
W3110
HMS174
BW25113
MG1655 (Genbank accession CP009685.1)
NCM3722
RR1
ETEC_H10407
SEC470
ST540 (Genbank accession CP007265.1)
ST540 (Genbank accession CP007390.1)
ST540 (Genbank accession CP007391.1)
ATCC_8739
PCN061
HS
KF1

Figure S2

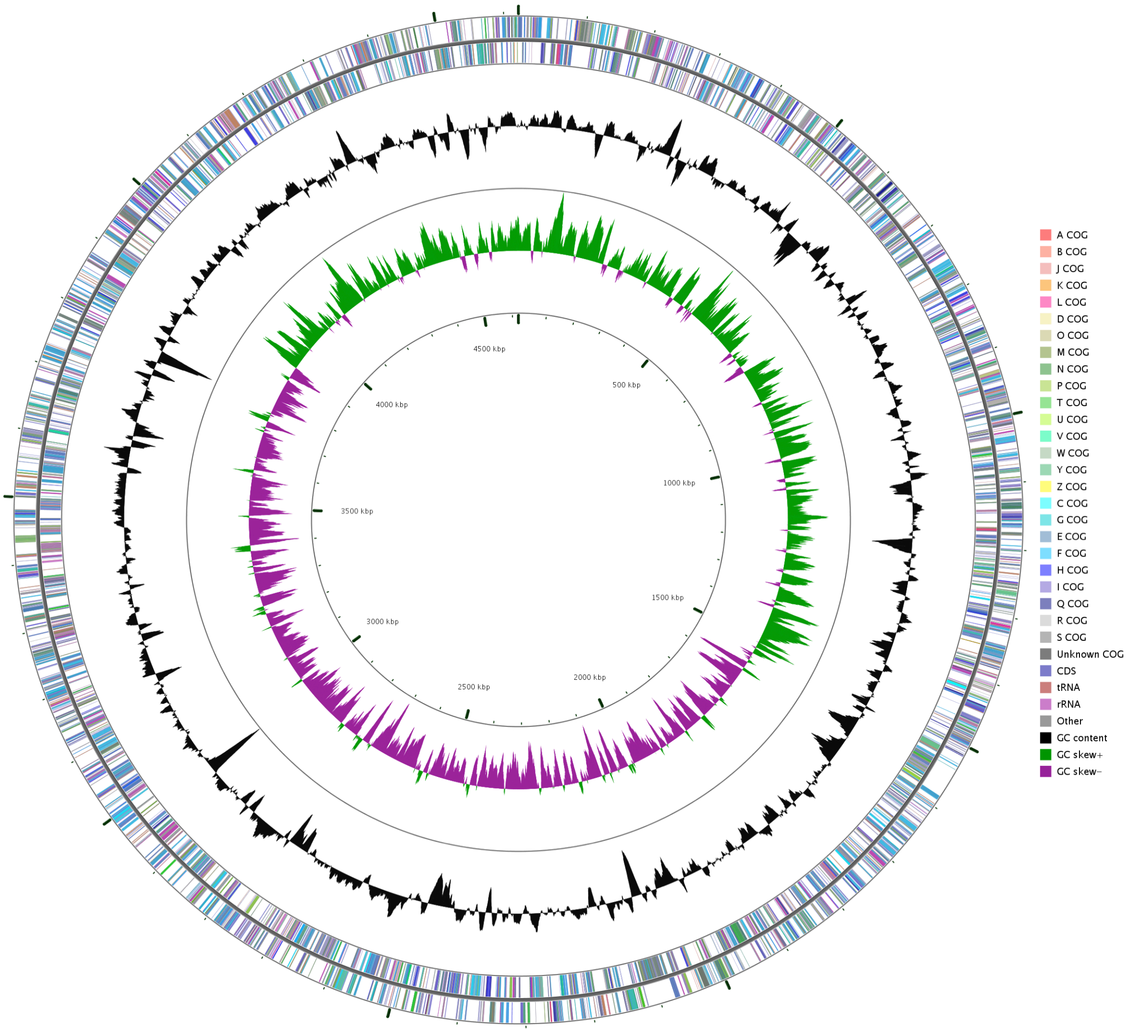

4500 kbp
500 kbp
4000 kbp
1000 kbp
3500 kbp
1500 kbp
3000 kbp
2000 kbp
2500 kbp
A COG
B COG
J COG
K COG
L COG
D COG
O COG
M COG
N COG
P COG
T COG
U COG
V COG
W COG
Y COG
Z COG
C COG
G COG
E COG
F COG
H COG
I COG
Q COG
R COG
S COG
Unknown COG
CDS
tRNA
rRNA
Other
GC content
GC skew+
GC skew–

Figure S3

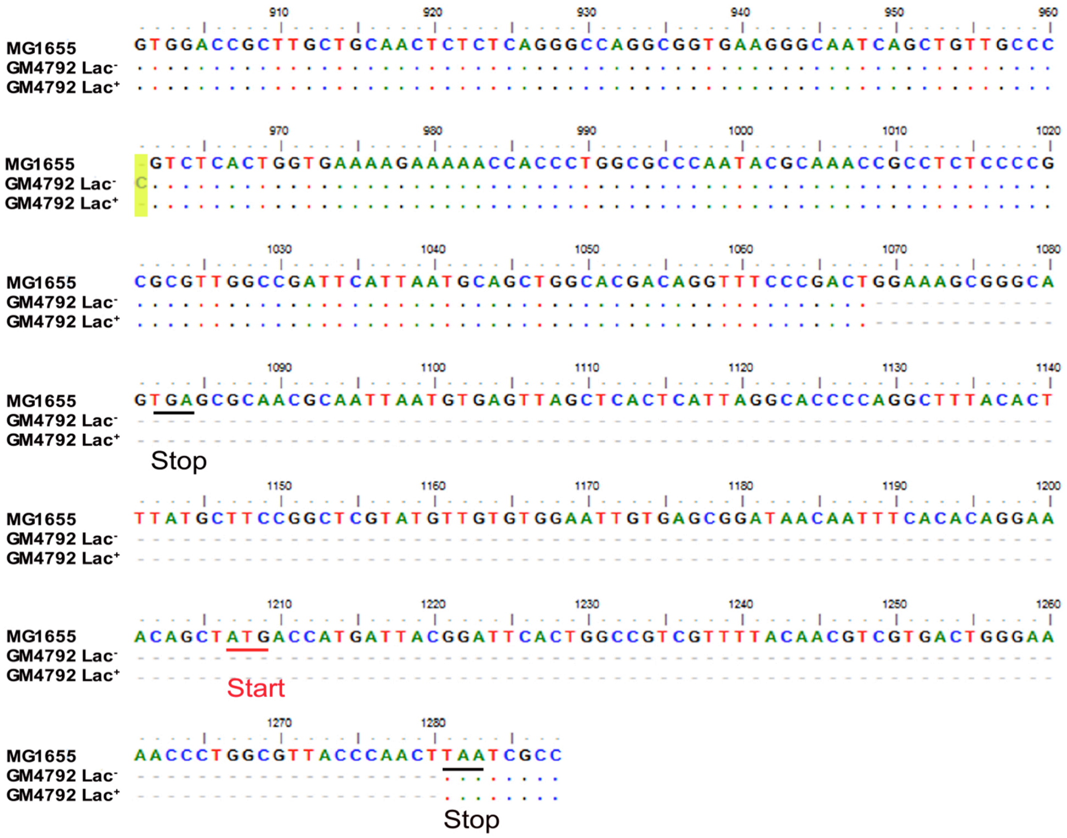

Figure S4
