# Supplementary material for: Genome sequences of two closely related strains of Escherichia coli K-12 GM4792
Source: Stand Genomic Sci. 2015 Dec 10;10:125. doi: 10.1186/s40793-015-0114-x (PMC4675052; doi:10.1186/s40793-015-0114-x)
Supplement: Additional file 2: — PCR experiment description. Table S8. The primer sequences for two GM4792 strains. Figure S5. The primer design for the large gap (~2,900 bps) in GM4792 Lac-. (PDF 124 kb) [file 40793_2015_114_MOESM2_ESM.pdf]

Genome sequences of two closely related strains of *Escherichia coli* K-12 GM4792

Yan-Cong Zhang[1], Yan Zhang[1*], Bi-Ru Zhu[1], Bo-Wen Zhang[1], Chuan Ni[1#], Da-Yong Zhang[1], Ying Huang[2], Erli Pang[1], Kui Lin[1]

[1]State Key Laboratory of Earth Surface Processes and Resource Ecology and MOE Key Laboratory for Biodiversity Science and Ecological Engineering, College of Life Sciences, Beijing Normal University, Beijing 100875, China

[2] State Key Laboratory for Infectious Disease Prevention and Control, and National Institute for Communicable Disease Control and Prevention, Chinese Center for Disease Control and Prevention, Beijing 102206, China

Corresponding author:
Kui Lin
College of Life Sciences, Beijing Normal University, 19 Xinjiekouwai Street, Beijing 100875, China
Email: linkui@bnu.edu.cn
Tel: 86 10 58805045
Fax: 86 10 58807721

[*] Current address: National Laboratory of Biomacromolecules, Institute of Biophysics, Chinese Academy of Sciences, Beijing 100101, China

[#] Current address: The second high school attached to Beijing Normal University, Beijing 100192, China

Additional PCR experiments

PCR experiments were carried out to close gaps in both genomes. Firstly, for small gaps, PCR primers were designed according to the sequences around each gap in the genomes. For the large gap (~2,900 bps) in GM4792 Lac^-, it is difficult for primers to amplify a complete product across this large gap. Fortunately, GM4792 Lac^- is so extremely closed to GM4792 Lac^+ that primers can be designed based on the corresponding homolog sequence in GM4792 Lac^+. More details are shown in Figure S5. All primer sequences are listed in Table S8. Secondly, Touchdown PCR amplifications were performed to increase specificity and sensitivity with genome DNA and primers in 25 μL reaction volume as two phases: touchdown phase with an initial annealing temperature of 58 °C, and then decrease the annealing temperature by 1 °C per cycle until 48 °C is reached for a total of 15 cycles; Phase 2 was a generic amplification stage of 25 cycles using the final annealing temperature 48 °C. After that, the products of PCR were loaded on a 1.5% agarose gel with TAE buffer, run at 120V for 30 min and stained with SYBR gold to test their purity. Each PCR product had only one band in the gel. Then, all PCR products were sequenced with Sanger. Finally, the amplified PCR sequences for small gaps were used to close gaps and perform corrections on the genome assembly. For the large gap (~ 2,900 bps) in GM4792 Lac^-, the four PCR sequences for this gap were assembled and used to close this large gap.

Tables

Table S8 – The primer sequences for two GM4792 strains.

Seq ID	Gap position[a]	Primer ID[b]	Primer sequence
GM4792 Lac^{+}	582,793	Lac^{+}_582793_F	TTATTGACCGAAAGGAGA
GM4792 Lac^{+}	582,793	Lac^{+}_582793_R	CCATTGCCAGAGTTGTAT
GM4792 Lac^{+}	736,048	Lac^{+}_736048_F	AATCAATTCCGCAATATC
GM4792 Lac^{+}	736,048	Lac^{+}_736048_R	ATGCCCCTCTAAACTAAG
GM4792 Lac^{+}	3,777,805	Lac^{+}_3777805_F	CCGCTGTAGAACTGAGGG
GM4792 Lac^{+}	3,777,805	Lac^{+}_3777805_R	CCGGATTATGGGAAAGAA
GM4792 Lac^{+}	4,523,274	Lac^{+}_4523274_F	TGGTAAGTAGCACGAAAT
GM4792 Lac^{+}	4,523,274	Lac^{+}_4523274_R	ACTGGATAGACCCAATAT
GM4792 Lac^{-}	737,380	Lac^{-}_737380_F	TGACAAGAGTCGCCGTAA
GM4792 Lac^{-}	737,380	Lac^{-}_737380_R	GGGACATCGCAAACAATA
GM4792 Lac^{-}	2,084,210	Lac^{-}_2084210_F1	ACTCGTGGAACAGCAGGGTA
GM4792 Lac^{-}	2,084,210	Lac^{-}_2084210_R1	CAGGGATGTTCTTGAAGAGGTG
GM4792 Lac^{-}	2,084,210	Lac^{-}_2084210_F2	AGCGTATCCCGACCTATC
GM4792 Lac^{-}	2,084,210	Lac^{-}_2084210_R2	TCTGCGGTCTTGAAAATA
GM4792 Lac^{-}	2,084,210	Lac^{-}_2084210_F3	AGAAAGGTTGCCAGCATA
GM4792 Lac^{-}	2,084,210	Lac^{-}_2084210_R3	CTGAATACTGGTAAGCAA
GM4792 Lac^{-}	2,084,210	Lac^{-}_2084210_F4	TGTTAATAGCCCCATCAG
GM4792 Lac^{-}	2,084,210	Lac^{-}_2084210_R4	CCGACTGACAATGATGTT

[a] The start position of gaps.

[b] xxx_F and xxx_R stand for the upstream and downstream primer, respectively.

Figures

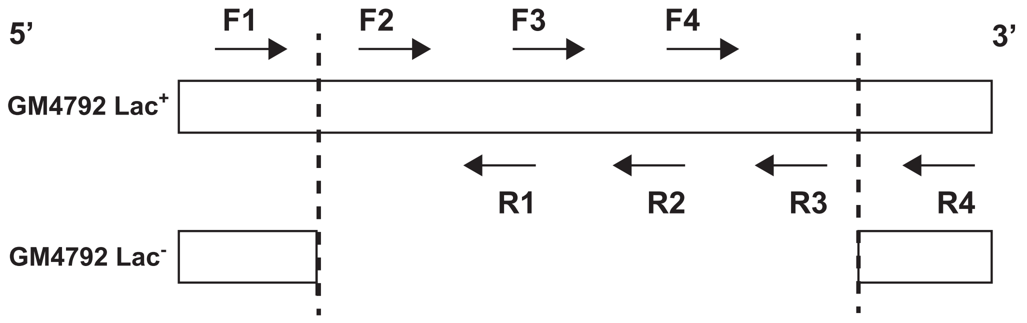

Figure S5 The primer design for the large gap (~ 2,900 bps) in GM4792 Lac$^-$.

As GM4792 Lac$^-$ is very closed to the GM4792 Lac$^+$, we designed the primers for the ~2,900 bps gap in GM4792 Lac$^-$ according to the corresponding homolog sequence in GM4792 Lac$^+$. Four pairs primers were designed and adjacent primers were overlapped for amplifying the full sequence.
