# Supplementary material for: Genome sequences of two closely related strains of Escherichia coli K-12 GM4792
Source: Stand Genomic Sci. 2015 Dec 10;10:125. doi: 10.1186/s40793-015-0114-x (PMC4675052; doi:10.1186/s40793-015-0114-x)
Supplement: Additional file 3: — Title of data: A document containing missing key taxonomic references. (PDF 96 kb) [file 40793_2015_114_MOESM3_ESM.pdf]

Genome sequences of two closely related strains of *Escherichia coli* K-12 GM4792

Yan-Cong Zhang[1], Yan Zhang[1*], Bi-Ru Zhu[1], Bo-Wen Zhang[1], Chuan Ni[1#], Da-Yong Zhang[1], Ying Huang[2], Erli Pang[1], Kui Lin[1]

[1]State Key Laboratory of Earth Surface Processes and Resource Ecology and MOE Key Laboratory for Biodiversity Science and Ecological Engineering, College of Life Sciences, Beijing Normal University, Beijing 100875, China

[2] State Key Laboratory for Infectious Disease Prevention and Control, and National Institute for Communicable Disease Control and Prevention, Chinese Center for Disease Control and Prevention, Beijing 102206, China

Corresponding author:

Kui Lin

College of Life Sciences, Beijing Normal University, 19 Xinjiekouwai Street, Beijing 100875, China

Email: linkui@bnu.edu.cn

Tel: 86 10 58805045

Fax: 86 10 58807721

[*] Current address: National Laboratory of Biomacromolecules, Institute of Biophysics, Chinese Academy of Sciences, Beijing 100101, China

[#] Current address: The second high school attached to Beijing Normal University, Beijing 100192, China

Missing Key taxonomic references

Annotation Summary

Name	Count	References	Comments
Enterobacteriales	1	G.M. Garrity, J.G. Holt: **Taxonomic Outline of the *Archaea* and *Bacteria***. *Bergey's Manual of Systematic Bacteriology* 2001, **1**: 155-166.	The order "Enterobacteriales" is Not Validly Published.
